# Aminophospholipids are signal-transducing TREM2 ligands on apoptotic cells

**DOI:** 10.1038/s41598-019-43535-6

**Published:** 2019-05-17

**Authors:** Keiro Shirotani, Yuma Hori, Ryohei Yoshizaki, Eri Higuchi, Marco Colonna, Takashi Saito, Shoko Hashimoto, Takashi Saito, Takaomi C. Saido, Nobuhisa Iwata

**Affiliations:** 1 Department of Genome-based Drug Discovery, Graduate School of Biomedical Sciences, Nagasaki University, Nagasaki, 852-8521 Japan; 2 Unit for Dementia Research and Drug Discovery, Graduate School of Biomedical Sciences, Nagasaki University, Nagasaki, 852-8521 Japan; 3 Department of Pathology and Immunology, Washington University School of Medicine, St. Louis, Missouri 63110 USA; 4 Laboratory for Cell Signalling, Department of Immunology, RIKEN Center for Integrative Medical Sciences, Kanagawa, 230-0045 Japan; 5 Laboratory for Proteolytic Neuroscience, RIKEN Center for Brain Science, Saitama, 351-0198 Japan

**Keywords:** Biological sciences, Biochemistry

## Abstract

Variants of triggering receptor expressed on myeloid cells 2 (TREM2) are associated with an increased incidence of Alzheimer’s disease, as well as other neurodegenerative disorders. Using a newly developed, highly sensitive reporter cell model, consisting of Jurkat T cells stably overexpressing a reporter gene and a gene encoding TREM2DAP12 fusion protein, we show here that TREM2-dependent signal transduction in response to apoptotic Neuro2a cells is mediated by aminophospholipid ligands, phosphatidylserine and phosphatidylethanolamine, which are not exposed on the intact cell surface, but become exposed upon apoptosis. We also show that signal-transducing TREM2 ligands different from aminophospholipids, which appear to be derived from neurons, might be present in membrane fractions of mouse cerebral cortex. These results may suggest that TREM2 regulates microglial function by transducing intracellular signals from aminophospholipids on apoptotic cells, as well as unidentified ligands in the membranes of the cerebral cortex.

## Introduction

Microglia, which are resident myeloid cells in the brain, provide acute and chronic surveillance of damage- and pathogen-associated signals as a part of the innate immune system^1^. The role of microglia in neurodegenerative disorders associated with chronic inflammation has been investigated extensively. In the Alzheimer’s disease (AD) brain, for example, reactive microglia are associated with amyloid plaques consisting of aggregated amyloid β peptide (Aβ)^2–4^. Aβ stimulates microglia and induces proinflammatory phenotypes that are thought to damage neurons and exacerbate disease progression^5,6^. On the other hand, microglia have protective roles against AD through phagocytosis of Aβ and apoptotic neurons^7,8^. Thus, proper regulation of diverse microglial functions should ameliorate disease progression. Genetic studies have identified several AD-associated risk factors expressed by microglia^9–12^. These include triggering receptor expressed on myeloid cells-2 (TREM2)^13,14^, whose homozygous mutations were found in certain types of neurodegenerative disorders, such as Nasu-Hakola disease and frontotemporal dementia-like syndromes^15–17^. Heterozygous mutations of TREM2 show a high odds ratio for the onset of AD^13,14^ as well as the other neurodegenerative disorders^18–20^. So far, R47H and R62H variants of TREM2 have been confirmed to present a significant risk for AD^21^, though other variants have not reached statistical significance, probably because of their low frequency. In addition, R47H mutation is correlated with increased tau levels in cerebrospinal fluid^22^ and increased phosphorylated tau levels around amyloid plaques^23^ in AD brains. Elevated expression of TREM2 gene is reported to ameliorate pathological phenotypes in AD models^24^. These studies highlight that functional alterations of TREM2 due to mutation contribute to the development of neurodegenerative disorders, possibly by loss of function^25,26^, and indicate that a better understanding of the role of TREM2 could provide new insights for drug discovery.

TREM2 is a type I transmembrane protein associated with DNAX-activating protein of 12 kDa (DAP12) in the transmembrane domain, and DAP12 has an immunoreceptor tyrosine-based activating motif (ITAM) in the cytosolic domain. TREM2 is therefore postulated to bind ligands at its extracellular immunoglobulin V-type domain and to transduce intracellular signals that regulate microglial functions such as cytokine production, migration, proliferation, phagocytosis, cell survival, synapse elimination, and compaction of amyloid plaques^23,27–34^. Notably, inflammatory cytokines are downregulated in Trem2-knockout mice^30,31,33^, and anti-Trem2 agonistic antibody induces inflammatory cytokines in the brain^35^. These results suggest that TREM2 has a role in the production of inflammatory cytokines *in vivo*. Interestingly, a functional interaction between TREM2 and familial AD gene presenilin 1 has been reported^36^. Proper signal transduction might be disturbed by the risk-associated variants, leading to increased incidence of neurodegenerative disorders. Many proteins or compounds that bind to the TREM2 extracellular domain have been reported, including phosphatidylserine (PS), phosphatidylethanolamine (PE), phosphatidylcholine (PC), sulfoglycolipid, apolipoproteins, low-density lipoprotein, high-density lipoprotein, heat shock protein 60, DNA, *E. coli*, apoptotic cells, and Aβ^31,37–54^. Among them, apoptotic cells^31,40^, normal cultured cells^39,40^, and oligomeric Aβ^53,54^ have been confirmed to induce TREM2-mediated intracellular signalling. However, the ligands expressed by apoptotic or normal cultured cells have not been identified, and it is not clear whether other signal-transducing ligands of TREM2 are present in the brain. In addition, further investigations are required to understand the different pathological effects of TREM2 variants on the signal transduction^50^.

In this work, we have developed a highly sensitive reporter cell model to monitor signal transduction from TREM2, in order to identify the TREM2 ligands on apoptotic and normal cultured cells and to search for other ligands in the brain that may be associated with increased incidence of AD. We found that the ligands expressed on apoptotic Neuro2a cells, as well as some normal cultured cells, are aminophospholipids. Finally we suggest that other TREM2 signal-transducing ligands are present in the membranes of mouse cerebral cortex.

## Results

### Establishment of reporter cell models for TREM2-dependent signal transduction

**Figure 1:**
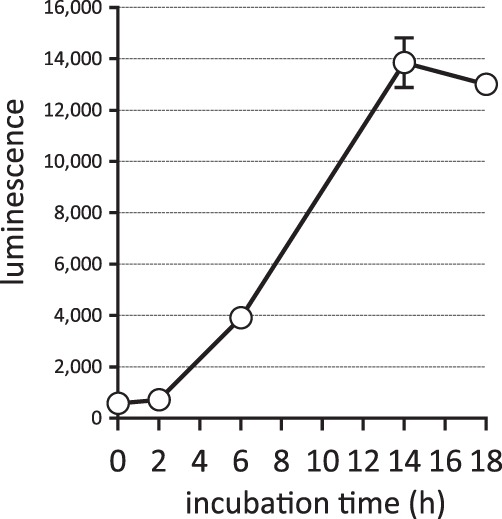
Time course of NAFT signal activation in Jurkat cell clones expressing TREM2DAP12 in the presence of apoptotic cells. Equal numbers of apoptotic cells and Jurkat reporter cell clones expressing TREM2DAP12 were incubated for the indicated times (hours) and the luminescence of the cell lysates was measured. Data are shown as means ± SD (n = 3). Where SDs are not shown, they are smaller than the symbols.

**Figure 2:**
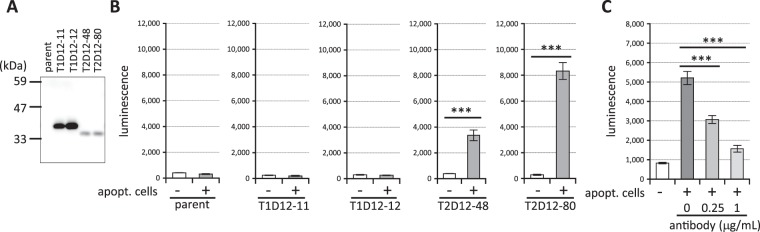
Establishment of TREM2-dependent reporter cells. (**A**) Lysates of Jurkat reporter cell clones expressing TREM1DAP12, TREM2DAP12 (11, 12, 48 and 80 indicate clone numbers) or parent reporter cells (parent) were immunoblotted by anti-DAP12 antibody. (**B**) Apoptotic cells (apoptot. cells) and the Jurkat reporter cell clones were incubated at a ratio of 1/2 and the luminescence of the cell lysates was measured. Data are shown as means ± SD (n = 3). Two-tailed Student’s t test was performed. (**C**) Apoptotic cells and Jurkat TREM2DAP12 reporter cell clones were incubated at a ratio of 1/10 together with anti-TREM2 antibodies and the luminescence of the cell lysates was measured. Data are shown as means ± SD (n = 3). One-way ANOVA with the Student-Newman-Keuls test was performed. ***p < 0.001.

**Figure 3:**
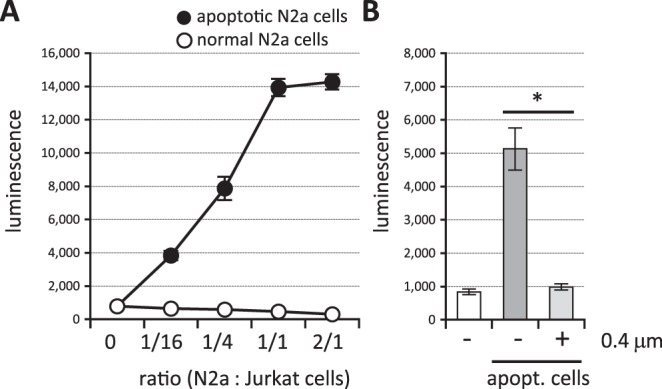
TREM2 ligands are exposed at the cell surface upon apoptosis. (**A**) Apoptotic (closed circles) or normal (open circles) Neuro2a cells and reporter cell clones expressing TREM2DAP12 were incubated with the indicated ratio of Neuro2a: the reporter cell numbers and the luminescence of cell lysates were measured. Data are shown as means ± SD (n = 3). Where SDs are not shown, they are smaller than the symbols. (**B**) Apoptotic cells and reporter cell clones expressing TREM2DAP12 were incubated at a ratio of 1/10 with or without culture inserts (0.4 μm pore size) and the luminescence of cell lysates was measured. Data are shown as means ± SD (n = 4). One-way ANOVA with the Student-Newman-Keuls test was performed. *p < 0.05.

To establish sensitive and specific reporter cell models to monitor TREM2 signal transduction, RAW264.7, 2B4, and Jurkat cells were stably transfected with reporter genes: nuclear factor of activated T cells (NFAT) promoter element and TREM2DAP12 fusion cDNA corresponding to TREM2 extracellular domain and DAP12 transmembrane and cytosolic domain^38,55,56^. RAW264.7 was chosen because it is a macrophage cell line having similar characteristics to microglia, such as phagocytosis and cytokine production. 2B4 and Jurkat cell lines were chosen because they are derived from T cells, which are the only cell type so far shown to transduce signals via TREM2^31,37,40^. These reporter cells were incubated with apoptotic Neuro2a cells to induce signal transduction via TREM2^40^ and luminescence or fluorescence of the cell lysates was measured. RAW264.7 reporter cells expressing TREM2DAP12 showed increased luminescence upon apoptotic cell treatment, but RAW264.7 cells not expressing TREM2DAP12 also showed increased luminescence, indicating that the signal is independent of exogenous TREM2DAP12 (not shown). On the other hand, 2B4 reporter cells expressing TREM2DAP12 showed a 2- to 3-fold increase of fluorescence upon apoptotic cell treatment, while the parent 2B4 cells (control) did not (not shown). More strikingly, Jurkat reporter cells expressing TREM2DAP12 showed a luminescence increase of more than 10-fold, while the parent cells did not (see Figs 1 to 3). These results confirmed that apoptotic cells express TREM2 ligand(s) that transduce an intracellular signal leading to NFAT activation^31,40^, and that T cell lines are suitable to study TREM2 signal transduction from apoptotic cells. In the following study, we used Jurkat reporter cells because of the high signal-to-background ratio.

We first investigated the time course of the NFAT activation by apoptotic cells. As shown in Fig. 1, luminescence of Jurkat reporter cell clones expressing TREM2DAP12 began to increase at 6 hours and reached a plateau at 14 hours when equal numbers of apoptotic cells and reporter cells were incubated. We then examined whether or not the signal is TREM2-dependent. Reporter cell clones expressing TREM2DAP12 or TREM1DAP12 or parent reporter cells (Fig. 2A) were incubated together with apoptotic cells, and the luminescence of the cell lysates was measured. As shown in Fig. 2B, two independent reporter cell clones expressing TREM2DAP12 showed increased luminescence upon apoptotic cell treatment, while the parent reporter cells and two independent reporter cell clones expressing TREM1DAP12 did not, even though the amounts of TREM1DAP12 proteins expressed in the reporter cells were much greater than those of TREM2DAP12 proteins (Fig. 2A). All of five independent TREM2DAP12-expressing cell clones that we established showed increased luminescence in response to the apoptotic cells (not shown), although the efficacy of signal transduction was different in each case. Moreover, anti-TREM2 antibody inhibited the reporter signal in a dose-dependent manner (Fig. 2C). These results indicate that the signal transduced in the TREM2DAP12-expressing reporter cells exposed to apoptotic cells is TREM2-dependent.

### TREM2 ligands expressed on apoptotic cells are aminophospholipids

Next, we examined the characteristics of the TREM2 ligands expressed in apoptotic cells. We incubated the reporter cells with apoptotic or normal cultured Neuro2a cells and measured the intensity of luminescence in the cell lysates. We found that apoptotic Neuro2a cells transduced the signal in a dose-dependent manner, while normal cultured Neuro2a cells transduced a signal similar to or weaker signal than that obtained with medium alone (Fig. 3A). When the reporter cells and apoptotic cells were cultured without direct contact (separated by a culture insert membrane with 0.4 μm pore size), the signal was almost completely blocked (Fig. 3B). These results suggest that TREM2 ligands are exposed on the cell surface upon apoptosis, and are not factors secreted from the cells.

**Figure 4:**
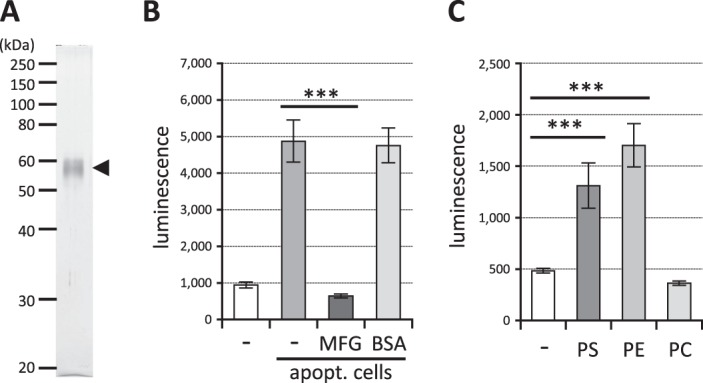
TREM2 ligands on apoptotic cells are aminophospholipids. (**A**) MFGE8-L D89E proteins were purified by anti-DDDDK antibody affinity column from culture media of HEK293 cells transfected with the MFGE8-L D89E cDNA. The purified proteins were electrophoresed and visualized by silver staining. (**B**) Apoptotic cells and reporter cell clones expressing TREM2DAP12 were incubated at a ratio of 1/10 together with 1 μg/mL MFGE8-L D89E or BSA as a control, and the luminescence of the cell lysates was measured. Data are shown as means ± SD (n = 3). One-way ANOVA with the Student-Newman-Keuls test was performed. (**C**) The reporter cell clones expressing TREM2DAP12 were cultured in wells coated with purified PS, PE or PC and the luminescence of the cell lysates was measured. Data are shown as means ± SD (n = 3). One-way ANOVA with the Student-Newman-Keuls test was performed. ***p < 0.001.

These characteristics of TREM2 ligands are reminiscent of aminophospholipids, PS or PE, which are localized in the inner leaflet of the plasma membrane in intact cells, but become exposed when the cells are triggered to undergo apoptosis^57^. Recombinant protein MFGE8-L D89E, which retains the binding domain to aminophospholipids but cannot bind phagocytes expressing integrins^58^, was purified to near homogeneity (Fig. 4A) and added to the culture medium of the reporter cells and apoptotic cells. As shown in Fig. 4B, MFGE8-L D89E completely inhibited the signal induced by apoptotic cells, while control protein (BSA) did not. This strongly indicated that the TREM2 ligands on the apoptotic cells are indeed aminophospholipids. In accordance with this observation, pure aminophospholipids PS and PE induced signal transduction in the reporter cells, while PC, which is located on the exterior of intact cells, did not (Fig. 4C). The EC50 values for PS and PE could not be determined because the reporter cells died before the luminescence reached a plateau in the presence of higher concentrations of the lipids (not shown).

### TREM2 ligands expressed on normal cultured cells are aminophospholipids

**Figure 5:**
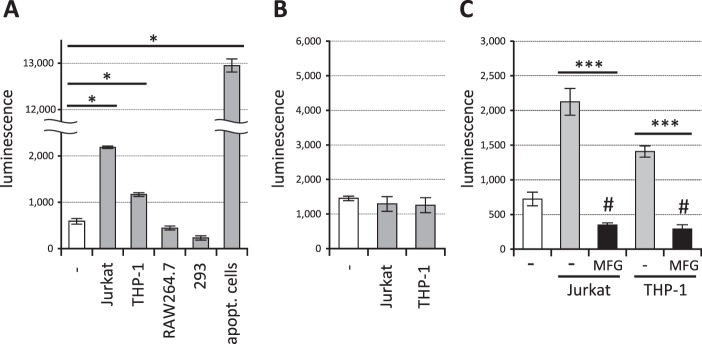
TREM2 ligands on normal cultured cells are aminophospholipids. (**A**) Various normal cultured cell lines and reporter cell clones expressing TREM2DAP12 were incubated at a ratio of 1/1 and the luminescence of the cell lysates was measured. Data are shown as means ± SD (n = 3). One-way ANOVA with the Student-Newman-Keuls test was performed. (**B**) Jurkat or THP-1 cell lines and reporter cell clones expressing TREM1DAP12 were incubated at a ratio of 1/1 and the luminescence of the cell lysates was measured. Data are shown as means ± SD (n = 3). (**C**) Jurkat or THP-1 cells and the reporter cell clones expressing TREM2DAP12 were incubated in a ratio of 1/1 together with 1 μg/mL MFGE8-L D89E and the luminescence of cell lysates was measured. Data are shown as means ± SD (n = 3). One-way ANOVA with the Student-Newman-Keuls test was performed. *p < 0.05; ***p < 0.001; ^#^*p* < 0.01 compared to the non-treated reporter cells (white column).

We then explored whether or not normal cultured cell lines express signal-transducing ligands of TREM2, because some cultured cell lines were shown to transduce NFAT signalling via TREM2^39,40^. We incubated the reporter cells with various cell lines and measured the luminescence. As shown in Fig. 5A normal cultured Jurkat and THP-1 cells induced signal transduction in TREM2DAP12-expressing reporter cells although the strength of luminescence was much less than that of apoptotic cells. This signal was TREM2-dependent, because TREM1DAP12-expressing reporter cells showed no signal transduction (Fig. 5B). When MFGE8-L D89E protein was added to the cultures, the signal was inhibited (Fig. 5C), suggesting that the TREM2 ligands on these normal cultured cells are also aminophospholipids, which might have become exposed during normal culture (see discussion).

### TREM2 ligands expressed in the brain are not aminophospholipids

**Figure 6:**
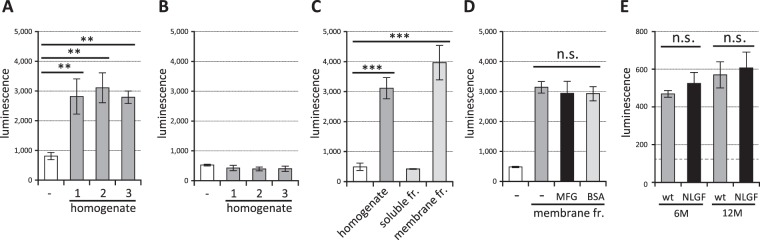
TREM2 ligands in membrane fractions of mouse cerebral cortex are not aminophospholipids. (**A**) Homogenates of cerebral cortices from three 5-month-old mice (no. 1 to 3) and reporter cell clones expressing TREM2DAP12 were incubated and the luminescence of cell lysates was measured. Data are shown as means ± SD (n = 3). One-way ANOVA with the Student-Newman-Keuls test was performed. (**B**) Homogenates of cerebral cortex from 5-month-old mice (no. 1 to 3) and reporter cell clones expressing TREM1DAP12 were incubated and the luminescence of cell lysates was measured. Data are shown as means ± SD (n = 3). (**C**) Homogenates, soluble fraction or membrane fraction of the cerebral cortex and reporter cell clones expressing TREM2DAP12 were incubated and the luminescence of the cell lysates was measured. Data are shown as means ± SD (n = 3). One-way ANOVA with the Student-Newman-Keuls test was performed. (**D**) Membrane fractions of the cerebral cortex and reporter cell clones expressing TREM2DAP12 were incubated together with 1 μg/mL MFGE8-L D89E or BSA and the luminescence of the cell lysates was measured. Data are shown as means ± SD (n = 3). **p < 0.01; ***p < 0.001; n.s. not significant. (**E**) Homogenates of cerebral cortices from 6- and 12-month wild-type and *App*^NL-G-F^ knock-in mice and reporter cell clones expressing TREM2DAP12 were incubated and the luminescence of cell lysates was measured. Luminescence of the reporter cells incubated with the buffer alone is shown as a broken line. Data are means ± SE (n = 3), and compared using Student’s t test. n.s. not significant.

Finally, we searched for TREM2 signal-transducing ligands in the brain. We incubated homogenates of cerebral cortices from young adult mice (5 months old) with reporter cells expressing TREM2DAP12 and measured the luminescence. We found that homogenates prepared from three independent mice increased the reporter activity (Fig. 6A) suggesting that the cerebral cortex contains ligand(s) that induce NFAT signalling via TREM2. This signal was TREM2-dependent, because reporter cells expressing TREM1DAP12 (Fig. 6B) or parent control cells (not shown) showed no signal. We then found that the ligands were present in the membrane fraction, not soluble fraction of the homogenates (Fig. 6C). Unexpectedly, the signal induced by the membrane fraction was not inhibited by MFGE8-L D89E (Fig. 6D); this result suggests that the ligands in the mouse cerebral cortical membranes are different from aminophospholipids. To examine if the ligands are expressed by astrocytes, microglia or neurons, we compared the TREM2 signal-inducing activity of cerebral cortices derived from wild-type and *App* knock-in mice carrying NL-G-F mutations (*App*^NL-G-F^)^59^, since the *App*^NL-G-F^ mice have increased numbers of astrocytes and microglia in an aging-dependent manner after 6 months^60^. However, we found no significant difference of TREM2 signal transduction between the two strains at 6 or 12 months (Fig. 6E) suggesting that the ligands might not be expressed by astrocytes or microglia.

## Discussion

We have established a highly sensitive and specific reporter cell model for TREM2 signal transduction in Jurkat T cells. So far, two cell lines, BWZ and 2B4 T cells, have been reported as TREM2 signal-transducing cell models^31,37,40^. Hsieh *et al*. transfected both TREM2/DAP12 cDNAs in BWZ cells expressing *lacZ* gene under the control of NFAT promoter, and Wang *et al*. introduced TREM2/DAP12 cDNAs in 2B4 cells stably expressing EGFP under the control of NFAT promoter. In the presence of apoptotic cells, the BWZ and 2B4 reporter cells showed about 4-fold and 1.2-fold increases of the reporter activity, respectively^31,40^. The Jurkat reporter cell model established in this study is even more sensitive, as the luciferase activity was increased by more than 10-fold in the presence of apoptotic cells (Figs 1 to 3). Possible reasons for this are that Jurkat cells may have greater amounts of signalling molecules required for NFAT signal transduction via TREM2, or that TREM2DAP12 fusion proteins are better able to transduce the signal than independently expressed TREM2 and DAP12. Another possibility might be that the *luc2P* reporter gene used in this study has humanized codons, which may be favorable for higher expression and reduced anomalous transcription. The *luc2P* gene also contains hPEST, a protein destabilization sequence, which allows luc2P protein levels to respond more quickly. A further advantage is that enzymatic detection of the *luc2P* is faster (5 min) than that of *LacZ*^40^. In addition, the procedure is simpler than sorting EGFP-expressing cells^31^, and is suitable for high-throughput screening of the agonists/antagonists. At the beginning of this study we tried to establish reporter cell models in RAW264.7 macrophage cell lines, but these cells transduced the signal irrespective of overexpression of TREM2DAP12. Endogenous expression of TREM2 and DAP12 in RAW264.7 cells^61,62^ might inhibit the signal from the overexpressed TREM2DAP12. As we and others have shown, T cell lines are suitable models for TREM2 signal transduction, probably because T cells express little endogenous TREM2 or DAP12 and share common signalling molecules with microglia, considering that T cell receptors and DAP12 have the same ITAM at the cytoplasmic domains.

The Jurkat reporter cells expressing TREM2DAP12 transduced the signal when incubated with apoptotic cells, while those expressing TREM1DAP12 did not (Fig. 2B), indicating the specificity of these reporter cells. We speculate that ligand recognition involves specific amino acid residue(s) in TREM2. Identification of the ligand-binding site in TREM2 would facilitate an understanding the interaction between TREM2 and the ligands, and would be helpful for drug development. The TREM2 antibody clone 237920 works as an antagonist of the NFAT signal via TREM2 induced by apoptotic cells (Fig. 2C). This may suggest that the epitope of the antibody is located near the binding site to apoptotic cells. Thus, mapping of the epitope might help to identify the ligand-binding site in TREM2. It would be interesting to see whether or not the epitope is identical to the reported PS-binding site in TREM2 extracellular domain^26^.

Apoptotic cells transduce the NFAT signal via TREM2^31,40^, while aminophospholipids such as PS and PE also transduce the signal via TREM2^31,48^. However, it has not been reported whether the TRME2 ligands in apoptotic cells are aminophospholipids. In this study we established for the first time that the TREM2 ligands in apoptotic cells are aminophospholipids by demonstrating inhibition of the signal with MFGE8 (Fig. 4B), an aminophospholipid-binding protein that inhibits the interaction between apoptotic cells and phagocytes^58^. This conclusion is supported by our findings that purified aminophospholipids (PS and PE), but not PC, transduce the signal (Fig. 4C), and that direct binding between reporter cells and apoptotic cells is required for signal transduction (Fig. 3B), because aminophospholipids are exposed on the cell surface after apoptosis, while PC is already exposed in healthy cells. Actually, Bailey reported that TREM2 binds to PS but not to PC^45^. However, other reports suggested that PC might induce signal transduction via TREM2^31,48^. The apparent contradiction may be due to the different reporter cell systems used (2B4 cells in their studies vs. Jurkat cells in this study). Bader Lange *et al*. reported that larger amounts of PS and PE are exposed on the surface of the synaptosomes in aged and AD model mouse brain^63^, and this may increase TREM2 signal transduction, leading to expression of microglial protective phenotypes. The increased incidence of AD associated with TREM2 R47H or R62H might be due to impaired protection of the TREM2 signal during aging or AD progression resulting from loss of function^50^. Although we still do not know exactly the target genes of TREM2 signal transduction responsible for the increased risk of AD, genes encoding factors involved in mTOR activation, which controls energetic and anabolic metabolism, might be candidates^64^. Further study will be needed to understand the link between TREM2 signal transduction and AD pathogenesis at the molecular level.

Our results indicate that the TREM2 ligands on normal cultured Jurkat and THP-1 cells are aminophospholipids (Fig. 5C). We hypothesize that small amounts of cells might die spontaneously during normal culture, resulting in exposure of previously inaccessible aminophospholipids. Since TREM2DAP12-expressing reporter cells are derived from Jurkat cells, aminophospholipids exposed on small amounts of the dying reporter cells might transduce the NFAT signal by themselves. Indeed, MFGE8 reduced the reporter activity to less than that of the normal cultured reporter cells (compare 3^rd^ and 5^th^ columns with 1^st^ column in Fig. 5C), supporting this hypothesis. Unknown TREM2 ligands on normal cultured cells that transduce the NFAT signal were previously reported^39,40^. Our results suggest that those unidentified ligands might be aminophospholipids.

Finally, we showed that TREM2 ligands that can specifically transduce intracellular signalling are present in the membrane fractions of 5-month-old mouse cerebral cortex, and that MFGE8 protein does not inhibit the signal (Fig. 6), suggesting that the signal-transducing ligands in the mouse brain are different from aminophospholipids. However, it is not clear why aminophospholipids, which should be present in the membrane fractions, cannot transduce the signal. One possible explanation would be that signal-transducing ligands in the brain may have a higher affinity for TREM2 than aminophospholipids, thus excluding the signal from aminophospholipids. Since nuclear and soluble fractions were removed from the membrane fractions, DNA and RNA can be excluded as candidates for the endogenous signal-transducing ligands. Subtle quantitative or qualitative alterations of the ligands might occur in the brain during aging, resulting in an increase in the incidence of AD. The TREM2 signal-inducing activities in the cerebral cortex from wild-type and *App*^NL-G-F^ knock-in mice were not significantly different (Fig. 6E), suggesting that the ligands might not be derived from astrocytes or from microglia, considering that the numbers of astrocytes and microglia are robustly increased after 6 months in *App*^NL-G-F^ knock-in mice^60^. Thus, the ligands might be expressed by live neurons. Identification of the ligands in the cortices will be an important next step for understanding the functional interaction between microglia and neurons, as well as the relevance of TREM2 to the development of neurodegenerative disorders. It would also be interesting to investigate whether AD-related variants such as R47H affect the binding and/or signal transduction from the ligands in the mouse cerebral cortex, and we are currently comparing the signal transduction characteristics of reporter cell clones expressing wild-type TREM2DAP12 and R47H variant.

In sum, we have developed a highly sensitive reporter cell model for signal transduction via TREM2 and showed that the TREM2 ligands on apoptotic and normal cells are aminophospholipids. The model should be useful to examine signal transduction by apolipoprotein E or oligomeric Aβ and to investigate the effects of TREM2 variants on the signal transduction.

## Methods

### cDNAs

Human TREM2^65^ and mouse MFGE8-L D89E^58^ cDNAs were as described. Human TREM1 and DAP12 cDNAs were obtained from Kazusa DNA Research Institute (Chiba, Japan) and Health Science Research Resources Bank (Osaka, Japan), respectively. cDNAs encoding the extracellular domain of TREM1 (amino acid residue 1-203) and TREM2 (amino acid residue 1-172) were amplified, fused with the transmembrane and cytosolic domain of human DAP12 (amino acid residues 30-113) with its stop codon by PCR, and inserted into the *Nhe*I and *Xho*I sites of pEBMulti-Neo TARGET tag-C (Fuji Film, Osaka, Japan). pGL4.30[*luc2P*/NFAT-RE/Hygro] vector (Promega, Madison, WI, USA) was used as a reporter gene, containing the luciferase reporter gene *luc2P* (*Photinus pyralis*) under the control of NFAT-responsive element.

### Culture cells

Jurkat human T cells, 2B4 mouse T cell hybridoma cells, and THP-1 human monocytes were maintained in RPMI1640 media (Fuji Film) supplemented with 10% fetal bovine serum (Sigma-Aldrich, St. Louis, MO, USA) and penicillin/streptomycin (Fuji Film). RAW264.7 mouse macrophages, HEK293 human embryonic kidney cells, and Neuro2a mouse neuroblastoma cells were maintained in DMEM (Fuji Film) supplemented with 10% fetal bovine serum and penicillin/streptomycin.

### Reporter cells

Jurkat cells were transfected with pGL4.30[*luc2P*/NFAT-RE/Hygro] in a NEPA21 super electroporator (Nepa Gene Corporation, Ltd., Chiba, Japan) and stably transfected clones were selected with 0.4 mg/mL hygromycin B (Fuji Film), followed by limiting dilution. Then, the cell clones were transfected with TREM2DAP12 (or TREM1DAP12) cDNA and stably transfected clones were selected with 1 mg/mL G418 (Fuji Film), followed by limiting dilution.

RAW264.7 cells were transfected with pGL4.30[*luc2P*/NFAT-RE/Hygro] using Lipofectamine2000 (Thermo Fisher Scientific, Waltham, MA, USA), and selected with 0.4 mg/mL hygromycin B. The cell clones were transfected with TREM2DAP12 cDNA and selected with 1 mg/mL G418.

2B4 cells stably transfected with enhanced green fluorescent protein (EGFP) gene under the control of NFAT-responsive element^66^ were transfected with TREM2DAP12 cDNA using a NEPA21 super electroporator and selected with 1 mg/mL G418.

### Western blot analysis

Cell lysates were extracted with lysis buffer (1% Triton-X 100, protease inhibitor cocktail (Complete^TM^, Roche Diagnostics, Indianapolis, IN, USA), 700 ng/ml pepstatin A (Peptide Institute, Osaka, Japan) in phosphate-buffered saline). Aliquots were electrophoresed on 10% polyacrylamide sodium dodecyl sulfate-gel and blotted onto polyvinylidene difluoride membrane (Hybond^TM^-P, Amersham Biosciences, Piscataway, NJ, USA). The membranes were probed with anti-DAP12 antibodies (Santa Cruz (Dallas, TX, USA)), followed by HRP-conjugated secondary antibodies IgG (Cell Signalling technology, Danvers, MA, USA). Immunoreactive bands were visualized with ImmunoStar LD (Fuji Film) and detected on a LAS-4000 mini (Fuji Film).

### Reporter assay

Neuro2a cells were treated with 100 nM staurosporin (Fuji Film) overnight to induce apoptosis, then washed and resuspended in the culture media. The apoptotic Neuro2a cells and 1 × 10^5^ Jurkat reporter cells were incubated at 37 °C in a ratio of 1/16 to 2/1 in 48-well plates for 14 hours, unless otherwise indicated. The cell lysates were incubated with One-Glo^TM^ reagent (Promega) in a 384-well plate for 5 min, and the luminescence was measured with a SPARK 20 M (Tecan, Männedorf, Switzerland). Normal cultured Neuro2a, Jurkat, THP-1, RAW264.7, and HEK293 cells were collected by pipetting or with TrypLE™ Select CTS™ (Thermo Fisher Scientific) and resuspended in the culture media after centrifugation. Where indicated, anti-TREM2 antibody (clone 237920, R&D Systems, Minneapolis, MN, USA), purified milk fat globule-EGF factor 8 (MFGE8)-L D89E, or bovine serum albumin (BSA) (Takara Bio, Osaka, Japan) was added to the culture media. Culture inserts with 0.4 μm pore membrane (Thermo Fisher Scientific) were used to block direct interaction between the apoptotic cells and reporter cells. Phosphatidylserine (PS), phosphatidylethanolamine (PE) and phosphatidylcholine (PC) (Sigma-Aldrich) were dissolved in methanol and coated on plates by drying before the reporter cells were seeded. Homogenates of cerebral cortices from wild-type and *App*^NL-G-F^ knock-in^59^ mice were prepared by homogenization in buffer (50 mM Tris-HCl buffer (pH 7.4), 150 mM NaCl, protease inhibitor cocktail, 0.7 ug/ml pepstatin A). The homogenates were centrifuged at 800 g for 10 min at 4 °C to remove nuclei and cell debris, and the post nuclear supernatants were centrifuged at 70,000 rpm in a TLA110 rotor (Beckman Coulter Diagnosis, Brea, CA, USA) for 29 min at 4 °C. The supernatants (soluble proteins) and resuspended membrane pellets were incubated with the reporter cells. All experiments conducted here were approved by the Ethics Committees of Nagasaki University.

### Preparation of MFGE8-L D89E protein

HEK293 cells were transfected with MFGE8-L D89E cDNA^58^ using Lipofectamine3000 (Thermo Fisher Scientific). After 24 hours, the medium was replaced with DMEM without fetal bovine serum and the cells were further cultured for 30 hours. MFGE8-L D89E protein in the culture media was purified with anti-DDDDK-tag mAb-Magnetic Beads (MBL, Aichi, Japan) and subjected to silver staining (Silver Stain MS kit (Fuji Film).

### Statistical analysis

Statistical analysis was done by SigmaPlot software, ver.14.0 (Systat Software Inc., San Jose, CA). To compare two groups, a two-tailed Student’s t test was employed after confirming equality of variances of the groups. To compare more than two groups, we used one-way ANOVA with the Student-Newman-Keuls test. *P* values of less than 0.05 were considered to be significant.
